# Supplementary material for: Moving Evidence into Practice by Advanced Practice Nurses in Hospitalization Wards. Protocol for a Multicentre Quasi-Experimental Study in Three Hospitals in Spain
Source: Int J Environ Res Public Health. 2020 May 16;17(10):3473. doi: 10.3390/ijerph17103473 (PMC7277768; doi:10.3390/ijerph17103473)
Supplement: Supplementary file 1 [file ijerph-17-03473-s001.pdf]

Supplementary appendix 1. Spanish validated Instruments

	Definition	*Subjects*
Practice Environment Scale Nursing Work Index (PES-NWI) (39).	Instrument used to evaluate factors related to nurses' satisfaction with their work, the quality of the care provided and the organisational characteristics of the hospital. A development of the original NWI, it is composed of 31 items and 5 subscales. The possible score ranges from 31 to 124 points. EFA (62.29% of total variance explained), CFA (chi2 = 673.1261, gl = 149, $p<0.0001$, RMSEA = 0.1996, SRMR = 0.0648).	Nurses from each of the hospital wards; Potential intervention wards.
Health Science Evidence Based Practice (HS-EBP) (40).	Transprofessional instrument used in health sciences for the self-assessment of personal abilities to implement EBP. HS-EBP measures the main factors external to the EBP process itself, which may influence its adoption, both from the individual standpoint, according to beliefs and attitudes, and from that of the organisation. It consists of 5 dimensions and 60 items. CFA (RMSEA = 0.049; 90%CI RMSEA = [0.047; 0.050]; CFI = 0.99).	Nurses from each of the control and intervention wards in the study.
Advanced Practice Nursing Competency Assessment Instrument (APNCAI) (41).	Instrument based on a comprehensive bibliographic search to compile and define the map of competencies that an APN must possess, within a construct composed of 8 factors-competency domains and 44 professional competencies. CFA (RMSEA = 0.05; 90%CI [0.05-0.06]: CFI = 0.99; SRMR 0.05).	Nurses receiving EBP training (APNs, support nurses and supervisors).

EFA, Exploratory factor analysis; CFA, Confirmatory Factor Analysis; RMSEA, Root Mean Square Error of Approximation; SRMR, Standardised Root Mean Square Residual; EBP, Evidence-based practice; CFI, Confirmatory fit index.
